# Caffeine: A Neuroprotectant and Neurotoxin in Traumatic Brain Injury (TBI)

**DOI:** 10.3390/nu17111925

**Published:** 2025-06-04

**Authors:** Bharti Sharma, George Agriantonis, Sarah Dawson-Moroz, Rolanda Brown, Whenzdjyny Simon, Danielle Ebelle, Jessica Chapelet, Angie Cardona, Aditi Soni, Maham Siddiqui, Brijal Patel, Sittha Cheerasarn, Justin Chang, Lauren Cobb, Fanta John, Munirah M. Hasan, Carrie Garcia, Zahra Shaefee, Kate Twelker, Navin D. Bhatia, Jennifer Whittington

**Affiliations:** 1 Department of Surgery, NYC Health and Hospitals—Elmhurst, New York, NY 11373, USA; agriantg@nychhc.org (G.A.); cheerass@nychhc.org (S.C.); hassanm14@nychhc.org (M.M.H.); carrie.garcia@nychhc.org (C.G.); shafaeez1@nychhc.org (Z.S.); bhatian1@nychhc.org (N.D.B.); harrisj20@nychhc.org (J.W.); 2 Department of Surgery, Icahn School of Medicine at Mount Sinai, New York, NY 10029, USA; 3 Department of Medicine, East Tennessee State University, Johnson City, TN 37614, USA; morozs@mail.etsu.edu (S.D.-M.);; 4 Department of Medicine, St. George’s University, Grenada, NY 11739, USA; rolandabrown95@yahoo.com (R.B.); debelle@sgu.edu (D.E.); jchapele@sgu.edu (J.C.); acardona@sgu.edu (A.C.); asoni1@sgu.edu (A.S.); msiddiq8@sgu.edu (M.S.); brij22.bp@gmail.com (B.P.); jchang3@sgu.edu (J.C.); lcobb@sgu.edu (L.C.); fanceesay@gmail.com (F.J.)

**Keywords:** Traumatic Brain Injury, caffeine, blunt trauma, penetrating trauma, clinical outcomes, neuroprotectant, neurotoxin

## Abstract

Caffeine is a weak, nonselective adenosine receptor antagonist. At low-to-moderate doses, caffeine has a stimulating effect; however, at higher doses, it can act as a depressant. It can function both as a neuroprotectant and a neurotoxin. In experimental Traumatic Brain Injury (TBI), administration of this psychoactive drug has been associated with beneficial or detrimental effects, depending on the dose, model, and timing. In a healthy brain, caffeine can enhance alertness and promote wakefulness. However, its consumption during late adolescence and early adulthood disrupts normal pruning processes in the context of repetitive moderate TBI (mTBI), leading to changes in dendritic spine morphology, resulting in neurological and behavioral impairments. Caffeine can potentially reduce TBI-associated intracranial pressure, oxidative stress, lipid peroxidation, cytotoxic edema, inflammation, and apoptosis. It can enhance alertness and reduce mental fatigue, which is critical for the cognitive rehabilitation of TBI patients. Additionally, caffeine positively affects immune cells and aids recovery post-TBI. Antagonizing adenosine receptors involved in controlling synaptic transmission, synaptic plasticity, and synapse toxicity can improve cognitive function. Conversely, studies have also shown that caffeine consumers report significantly higher somatic discomfort compared to non-consumers. This review aims to explore various studies and thoroughly examine the positive and negative roles of caffeine in TBI.

## 1. Introduction to Traumatic Brain Injury (TBI)

Traumatic Brain Injury (TBI) is increasingly recognized as a significant and escalating public health concern, impacting millions globally. TBI is characterized by the physiological or structural disruption of cerebral homeostasis, resulting from either penetrating injury or blunt force trauma to the cranium [1]. The pathophysiology of TBI can be classified into primary and secondary brain injuries, depending on whether the inciting trauma directly or indirectly disrupts cerebral structures [2]. Primary brain injury refers to the immediate damage that occurs at the time of impact, typically within milliseconds to seconds [2]. These injuries are further categorized into focal and diffuse forms [2]. Focal primary brain injuries include subarachnoid hemorrhages, subdural hemorrhages, epidural hemorrhages, intracerebral hemorrhages, cerebral contusions, brain parenchymal lacerations, coup-contrecoup injuries, and intracerebral as well as intracerebellar hematomas [2]. Secondary brain injury is characterized by a series of disturbances in cerebral homeostasis, including alterations in systemic parameters such as blood pressure, oxygenation, and intracranial pressure, which evolve after an initial acute insult to the brain or spinal cord [3].

The clinical manifestations of TBI exhibit variability depending on the site, severity, and specific subtype of TBI incurred by the patient [4,5]. General symptoms of TBI can be categorized into subgroups, including global neurological symptoms, focal neurological deficits, signs indicative of heightened intracranial pressure, and abnormal posturing [4,5]. Global neurological symptoms encompass headache, amnesia, loss of consciousness, or altered consciousness accompanied by disorientation, confusion, or potentially a lucid interval [4,5]. Focal neurological deficits typically correlate with the specific anatomical region of brain involvement, yielding symptoms such as ataxia, dysmetria, dysarthria, cranial nerve palsies affecting visual and auditory acuity, sensory deficits, and hemiparesis or hemiplegia contingent on injury severity [4,5].

In the United States, TBI contributes to 30% of all injury-related fatalities [6]. According to the Centers for Disease Control and Prevention (2023) [7], approximately 2.8 million individuals sustain TBI annually, a figure comparable to the population of Mississippi [6]. In 2020, there were 214,110 hospitalizations directly attributed to TBI, and in 2021, 69,473 deaths were recorded due to TBI [6], among different genders, males exhibit a significantly higher propensity for hospitalization (age-adjusted rate: 79.9 vs. 43.7) and mortality (age-adjusted rate: 28.3 vs. 8.4) due to TBI, with a twofold and threefold greater likelihood, respectively, compared to females [6]. This disparity is attributed to the greater engagement of males in high-risk activities, predisposing them to brain injury [6]. Globally, TBI impacts an estimated 69 million individuals annually, a figure roughly equivalent to twice the population of California, underscoring the magnitude of this public health concern worldwide [6].

Numerous risk factors are linked to TBI, encompassing demographic variables such as age and gender, occupational hazards, substance abuse patterns, and engagement in high-contact sports or hazardous activities [8]. These factors collectively contribute to the varying incidence and severity of TBI observed across populations, highlighting the multifactorial nature of TBI risk assessment and prevention strategies [8]. In terms of age demographics, epidemiological data consistently indicate that elderly adults and very young children exhibit heightened susceptibility to TBI, primarily attributable to increased incidences of falls and accidents within these age brackets [6,8]. Substance abuse, encompassing both drug and alcohol misuse, further escalates the risk of accidents, predisposing individuals to TBI and underscoring the critical importance of preventive measures and intervention strategies aimed at mitigating TBI incidence in high-risk populations [6,8]. Finally, individuals engaged in high-contact sports, activities prone to significant falls, or tasks involving heavy lifting inherently elevate their risk of experiencing TBI [6,8]. These pursuits necessitate heightened awareness of injury prevention strategies and comprehensive management protocols to mitigate the incidence and severity of TBI associated with such activities [6,8]. Therefore, in this review, we present a literature search that provides insights into the various roles of caffeine in TBI.

## 2. Pharmacology of Caffeine

**Figure 1:**
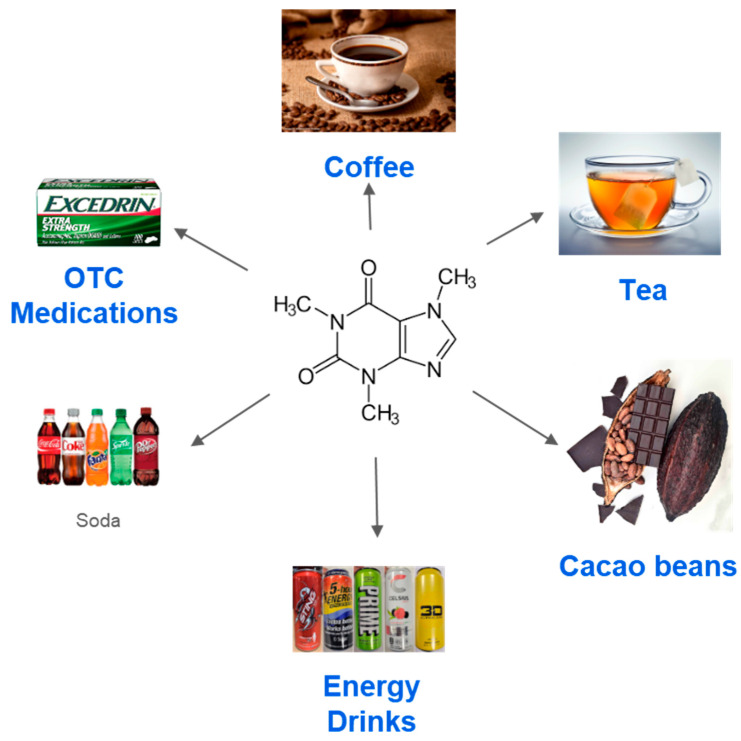
It shows various sources of caffeine. Caffeine, also known as C_8_H_10_N_4_O_2_, can be natural or synthetic. Some of the natural sources of caffeine include coffee, tea, and cacao beans. Alternatively, caffeine can be found synthetically in energy drinks, soda, and over-the-counter pain medications.

Caffeine (C_8_H_10_N_4_O_2_), also known as 1,3,7-trimethylxanthine, is a naturally occurring alkaloid that belongs to the methylxanthine class with associated bioactive compounds such as polyphenols [9]. It is one of the most used psychoactive stimulants worldwide. As shown in Figure 1, it can be found in a variety of foods and beverages, including coffee, tea, cacao beans, energy drinks, soda, and certain over-the-counter medications [10]. Caffeine’s structure consists of a xanthine core—a purine ring system—substituted with three methyl groups at the 1, 3, and 7 positions. This structure closely resembles that of adenosine, a key neuromodulator in the central nervous system [11,12]. It is also closely structurally related to other natural xanthines, such as theobromine, theophylline, and paraxanthine [12,13].

Once orally ingested, caffeine is rapidly absorbed from the gastrointestinal (GI) tract and reaches its peak plasma concentration within 30–60 min [1]. Due to its dual lipophilic and hydrophilic nature, caffeine crosses cell membranes, including the blood–brain barrier, which gives rise to its central nervous system (CNS) stimulant effects [14]. Caffeine metabolism in humans is performed through the demethylation reaction, converting caffeine (1,3,7-trimethylxanthine) to paraxanthine (1,7-dimethylxanthine), theobromine (1-demethylated product), and theophylline (7-demethylated product) [15]. This reaction occurs in the liver and is facilitated by the activity of CYP1A2, a cytochrome P450 isoform [16]. The half-life and clearance of caffeine are dependent on many outside factors, including environment, genetics, age, medications, smoking status, etc. [14]. In healthy adults, the half-life of caffeine ranges from about 3 to 5 h [14]. Caffeine increases the bioavailability of some drugs, such as aspirin, ergotamine, and levodopa, by decreasing gastric pH, which helps accelerate the absorption rate of the drugs mentioned [17].

Caffeine is known for its stimulatory, antioxidant, anti-inflammatory, and pain-relieving properties [18]. Caffeine consumed in moderate amounts (generally up to 400 mg per day for healthy, non-pregnant and non-lactating adults and up to 200 mg per day for those who are pregnant or lactating) is associated with several health benefits. These include increased alertness, reduced fatigue, enhanced cognitive performance (such as attention, vigilance, and reaction time), and improved physical performance, especially in endurance and high-intensity activities [11,19]. FDA-approved indications for caffeine include IV caffeine to treat apnea of prematurity and oral caffeine for restoring mental alertness or wakefulness [9]. For apnea, IV caffeine citrate is used, which contains 20 mg of caffeine citrate per milliliter [20]. Caffeine has also been used off-label for the treatment of migraines as well as in athletes as a performance enhancer [9]. Since caffeine produces a vasoconstrictive effect, which leads to decreased cerebral blood flow, it may help in acute settings of headaches [21]. Migraine sufferers should limit their caffeine intake to 200 mg to avoid risking the worsening of symptoms [21]. Various caffeine-related targets and pathways are listed in Table 1 of this paper.

Additionally, previous studies have explored the neuroprotective effects of caffeine in association with neurodegenerative diseases and stroke [22,23,24,25]. One 2020 meta-analysis by Hong et al. reviewed 13 studies and found that individuals who regularly consume caffeine presented a significantly lower rate of Parkinson’s disease (PD) risk and progression in both healthy individuals and those diagnosed with PD [22]. Another meta-analysis published by Li et al. (2024) [23] investigated the association between caffeine intake and risk of dementia and Alzheimer’s disease (AD) in 38 cohort studies. The investigators found that increased tea consumption was associated with a decreased risk of dementia and AD, while a non-linear relationship was found between coffee and dementia [23]. A 2022 study by Ruggiero et al. found that consumption of caffeine at doses of 3–5 mg/kg has been associated with a reduced risk of Alzheimer’s disease and Parkinson’s Disease [26]. Several large-scale meta-analyses published in the last few years have also demonstrated that moderate coffee consumption is associated with a reduced risk of stroke, particularly ischemic stroke [24,25].

It has been shown that caffeine can protect cells by reducing damage caused by oxidative stress and reactive oxygen species (ROS), giving it its antioxidant properties [27]. Animal studies in rats have demonstrated that caffeine can reverse oxidative stress and inflammation caused by certain compounds [28]. In TBI, a major concern is neuroinflammation and toxicity, so by using nano coffee particles, significant improvements in behavior, proteins, and dendritic cells can be seen during treatment for TBI [13].

**Table 1:** This table summarizes the numerous impacts of caffeine on various signaling pathways, genes, and proteins in TBI in animal studies.

Impact	Mechanism	Pathway	Genes/Proteins
Neuroprotection [29,30]	Reduces cell death and improves cognitive function post-injury [31]	Activates adenosine receptors, leading to downstream neurotransmitter release and neuroinflammatory pathways [30]	Modulates expression of genes involved in inflammation, like TNF-α and IL-1β, and neuronal survival, like BDNF and NGF [32]
Anti-inflammatory [31]	Inhibits microglial activation and reduces the release of pro-inflammatory cytokines [31]	Downregulates NF-κB signaling and modulates MAPK pathways [31]	Alters expression of inflammatory mediators (e.g., TNF-α, IL-1β) [30,31]
Improvement of Cognitive Function [33]	Enhances synaptic plasticity and neurotransmitter systems (e.g., acetylcholine) [33]	Increases intracellular calcium by inhibiting cAMP phosphodiesterase and directly activating ryanodine receptors [29,32,34]	Upregulates neurotrophic factors (e.g., BDNF, NGF) [29,34]

## 3. Numerous Effects of Caffeine in TBI

### 3.1. Caffeine as a Neuroprotectant and Neurotoxin

Caffeine provides neuroprotection through long-term upregulation of adenosine A1 receptors or acute inhibition of A2a receptors. Research on adenosine receptor knockout (KO) mice indicates that many of caffeine’s acute effects might be mediated by the adenosine A2a receptor (A2AR) [33,35]. Blocking the A2a receptor reduces brain damage after a TBI [36]. Under normal circumstances, adenosine levels are regulated for receptor interaction, but, during severe metabolic stress like TBI, adenosine surges and excessively stimulates A1 and A2A receptors [37,38].

Caffeine has also been demonstrated to play a crucial role in protecting the brain against various types of damage, including neurotoxicity, seizures, and cognitive dysfunction, through its antioxidant mechanisms [36]. A single acute dose of caffeine administered after the injury can prevent lethal apnea, regardless of prior chronic caffeine exposure [36]. In an experiment performed by Lusardi et al. (2012) [37], involving the use of caffeine in adenosine receptor blockade and knockout mice, post-injury caffeine treatment rapidly restored spontaneous breathing [36]. In a follow-up study performed by Lusardi et al. (2020) [38], administering a single acute bolus of caffeine (25 mg/kg) to rats 10 s after a severe TBI nearly completely prevented lethal apnea in conditions where 40% of the animals would have otherwise succumbed [36].

The favorable effects of chronic caffeine consumption are likely due to the upregulation of adenosine A1 receptor expression [39]. In an experiment conducted by Ning et al. (2019) [40] investigators studied the contribution of different methods of caffeine application on outcomes in whole-body blast injury models (WBBI) in mice and found that the chronic caffeine group showed alleviated neurological deficits and higher locomotor activity 24 h post-trauma compared to the acute caffeine and water groups [39]. In another preclinical study, administering caffeine to mice for three weeks significantly protected against neuro damage from a cortical contusion injury, whereas a single dose of caffeine in the same model had no effect [41,42].

Caffeine administration has been shown to have beneficial effects on the central nervous system against various cerebral insults, including acute brain damage such as TBI, in both experimental animals and clinical patients. Caffeine administered before injury is likely to impact the entire brain, while caffeine perfusion into the injured brain may be concentrated in the most severely affected areas [38]. The dose–response results after diffuse injury show that a 25 mg/kg caffeine treatment in rats significantly reduces acute mortality by 75% without impairing motor function, indicating that quality of life can be maintained with caffeine intervention following diffuse injury [36]. Clinical studies found that there is a significant association between a CSF caffeine concentration of at least 1 mmol/L (194 ng/mL) and a favorable 6-month outcome [35].

Another clinical study found that giving 2.5 mg/kg/day of caffeine for up to a month after injury improved consciousness and performance in children and adolescents with moderate TBI [43]. In a multicenter prospective study on patients with TBI and intracranial injury, a significant association between serum caffeine concentrations from 0.01 to 1.66 µg/mL and favorable functional recovery at the 6-month follow-up was observed [44]. Data suggest that injecting coffee extracts after a TBI may effectively reduce cognitive deficits and improve overall neuronal health and recovery [45]. Caffeine’s stability at room temperature makes it suitable for emergency kits, and intramuscular delivery could be a viable administration method in human trauma settings [38]. Some of the neuroprotective effects of caffeine are shown in Figure 1 of this paper.

Caffeine’s role as a neurotoxin in the context of TBI is linked to its interference with the brain’s natural protective mechanisms [46]. TBI disrupts the delicate balance of neurotransmitters and cellular homeostasis in the brain, leading to excitotoxicity, inflammation, and oxidative stress [14]. Following TBI, adenosine, a neuromodulator with anti-inflammatory and neuroprotective properties, accumulates to mitigate neuronal damage, but caffeine antagonizes adenosine receptors, impairing this protective effect [17]. This antagonism can exacerbate excitotoxicity and increase glutamate release, further contributing to neuronal injury and oxidative stress in the already vulnerable post-TBI brain [47]. By blocking A1 and A2A receptors, caffeine exacerbates neuronal stress and increases excitotoxic damage, particularly during periods of recovery after injury [33]. Caffeine’s ability to increase metabolic demands in neurons may intensify cellular energy deficits in the already compromised brain, potentially leading to impaired recovery and heightened neuronal damage [21].

Although caffeine can be neuroprotective, a study investigating the influence of caffeine on brain function, swelling, and permeability found that it significantly worsens neurological deficits and increases mortality in rats after an induced head injury [36]. The harmful effects of immediate caffeine consumption are likely due to the blockade of A1 receptors, supported by evidence showing that TBI in mice lacking A1 receptors resulted in fatal status epilepticus [36]. In an experiment conducted by Ning et al. (2019) [40], researchers studied the contribution of different methods of caffeine application on outcomes in whole-body blast injury models (WBBIs) in mice. They found that mortality was significantly higher in mice with acute and chronic caffeine treatment than in mice in the caffeine withdrawal and water groups [39]. In vitro studies show that the stretch injury of neurons reduces caffeine’s impact on calcium-induced calcium release, implying that pre-injury caffeine in caffeine-naïve neurons adds to the injury [36]. In a 2020 study by Christensen et al., it was found that caffeine consumption during late adolescence and early adulthood disrupts normal pruning processes in the context of repetitive moderate TBI (mTBI), leading to changes in dendritic spine morphology resulting in neurological and behavioral impairments [48].

While previous studies have highlighted the impact of adolescent caffeine exposure on dendritic spine morphology following TBI, emerging evidence suggests that caffeine also induces significant epigenetic modifications during this critical developmental period, potentially influencing TBI outcomes. Chronic caffeine consumption during adolescence has been shown to alter the expression of genes involved in DNA methylation, such as DNA methyltransferase (Dnmt1) and DNA methyltransferase 3a (Dnmt3a), in brain regions associated with cognition and stress response. These enzymes are crucial for establishing and maintaining DNA methylation patterns, which regulate gene expression without altering the DNA sequence. Disruptions in DNA methylation during adolescence can lead to long-lasting changes in gene expression, potentially affecting neural plasticity and recovery mechanisms following TBI [49].

Furthermore, caffeine intake has been associated with modifications in histone acetylation, another key epigenetic mechanism. Specifically, caffeine consumption can influence the acetylation status of histone H3 at lysine 27 (H3K27), a marker associated with active gene transcription. Alterations in H3K27 acetylation have been linked to changes in the expression of genes involved in synaptic function and neuronal signaling. These epigenetic changes may prime the brain for altered responses to injury, potentially affecting the severity and recovery trajectory of TBI experienced during or after adolescence [50].

**Figure 2:**
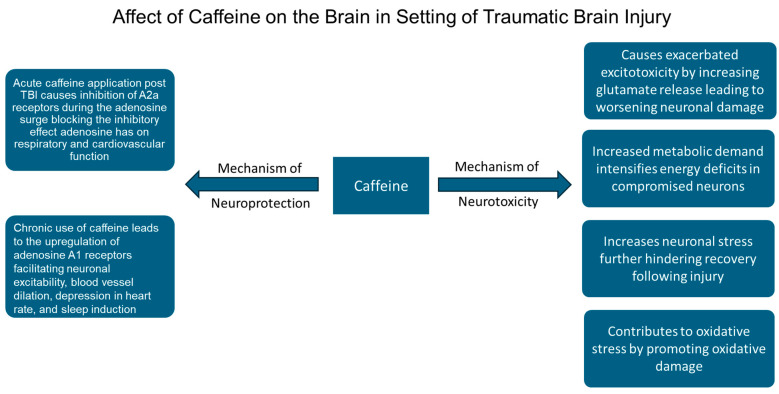
Effect of caffeine on the adenosine receptors to mediate neuroprotection and neurotoxicity in TBI.

Some of the neurotoxic effects of caffeine are illustrated in Figure 2 of this paper.

#### Caffeine as an Antioxidant and Anti-Inflammatory Pathway Independent of Adenosine

Although caffeine primarily exerts its pharmacological effects via antagonism of adenosine receptors, emerging evidence highlights several adenosine-independent mechanisms, particularly involving antioxidant and anti-inflammatory pathways. Caffeine has been shown to possess direct antioxidant properties, including the scavenging of reactive oxygen species (ROS) and the inhibition of oxidative damage to lipids, proteins, and nucleic acids. In addition, caffeine modulates inflammatory signaling cascades, notably through the suppression of NF-κB and MAPK pathways, which leads to the inhibition of NLRP3 inflammasome activation, a critical driver of neuroinflammation in traumatic brain injury (TBI) [31].

These anti-inflammatory effects are not mediated by adenosine receptor blockage, suggesting alternative molecular targets. Experimental studies in models of non-alcoholic steatohepatitis (NASH) and neurodegenerative diseases have demonstrated that caffeine downregulates toll-like receptor 4 (TLR4)-mediated signaling, a key initiator of innate immune responses. By extrapolation, similar mechanisms may be relevant in TBI, where microglial activation and cytokine release, such as release of IL-6 and IL-1β, contribute to secondary neuronal injury. Through the attenuation of these inflammatory mediators, caffeine may play a neuroprotective role by limiting tissue damage and supporting neuronal survival [31].

### 3.2. Effects of Caffeine on a Healthy Brain and an Injured Brain

#### 3.2.1. Healthy Brain

Caffeine enhances cognitive performance, boosts alertness, and promotes wakefulness [50]. A 75 mg dose of caffeine can improve reaction time and enhance visual and sustained attention, particularly during long, demanding tasks [51]. A 2020 UK study performed by Cornelis et al. [52] investigated the impact of recent caffeine drinking on cognitive ability, accounting for lifestyle and genetic factors that impact caffeine metabolism. The study included up to 434,900 participants in the UK Biobank aged 37–73 years. Recent caffeine consumption was associated with higher performance on reaction time tasks but lower performance on fluid intelligence, pairs matching, and prospective memory tasks [52]. However, more recent, smaller-scale studies have shown that the effects of caffeine on cognitive function and brain activity in healthy individuals vary based on dosage, chronicity of intake, and genetic factors [53,54,55]. For example, Ajjimaporn et al. (2022) studied the effects of 50 mg of caffeine on 25 healthy men and found that low-dose caffeine consumption significantly decreased alpha wave activity and improved working memory performance [53]. A 2023 randomized double-blind placebo-controlled trial of 20 healthy, non-smoking individuals by Lin et al. demonstrated that daily caffeine intake increased error rates and reaction times in working memory tasks compared to placebo, suggesting that chronic caffeine consumption may impede cognitive performance despite increased cerebral metabolic demands [54], thus highlighting the variability when considering caffeine dosage and chronicity, and suggesting that individual genetic variability may play a large role in caffeine’s specific effects on the brain. In a 2023 systematic review of 15 randomized control studies, six cross-sectional studies, and one genome-wide association study, Kapellou et al. demonstrated that polymorphisms in genes such as *CYP1A2* and *ADORA2A* were found to influence individual responses to caffeine [55]. Variants of the *ADORA2A* gene influence caffeine-induced anxiety. Regular caffeine consumption can lead to tolerance to its anxiety-inducing effects through central mechanisms [56].

Caffeine influences sleep by delaying sleep onset, reducing total sleep time, and increasing light sleep phases [57]. These effects can occur with doses as low as 100 mg. These disruptions persist even when caffeine is consumed in the morning, leading to less overall sleep and a longer time to enter stage 2 sleep [56]. However, habitual caffeine users may not experience these sleep disruptions as severely. Individual sensitivity to caffeine’s effects on sleep varies significantly [58]. Sensitive individuals may experience nearly twice the incidence of insomnia when consuming caffeine compared to those who are less sensitive [59].

#### 3.2.2. Injured Brain

Caffeine can help alleviate fatigue, hypersomnia, and reduced energy levels commonly experienced by TBI [60,61]. An animal model conducted by Lusardi et al. (2012) of severe TBI revealed that caffeine treatment prevents lethal apnea and decreases epileptiform EEG activity while also demonstrating no adverse effects on neuromuscular behavior or histological outcomes [37].

Stimulants such as caffeine improve cognitive performance, auditory vigilance, and reaction times [62]. However, they can hinder the brain’s ability to adapt and disrupt normal sleep, both of which are critical for recovering from TBI [63]. An animal study by Yamakawa et al. (2017) examined the impact of adolescent caffeine use on recovery from repetitive mild TBI (RmTBI) and post-traumatic symptoms [64]. In male rats, combining caffeine with RmTBI exacerbated negative outcomes in tests like open field and forced swimming, compared to either treatment alone [62]. Females showed similar disruption from RmTBI and caffeine exposure in tests, including open field [64].

Li et al. (2008) performed a study aimed at assessing the impact of acute and chronic caffeine treatment on TBI using the weight-dropped model in mice [65]. It was found that chronic caffeine treatment has been observed to decrease cell apoptosis in the brain following TBI [64]. A rat study by Lusardi et al. (2020) found that a single intraperitoneal dose of caffeine administered immediately after severe TBI restored normal breathing patterns and prevented lethal injury-induced apnea [38]. Researchers investigated the potential applicability of caffeine treatments in humans with varying histories of caffeine consumption, testing acute administration post-injury with different doses and patterns [64]. They found that administering a single dose of caffeine after TBI could potentially prevent fatal outcomes in various scenarios [64]. A clinical study used an animal model completed by Sanjakdar et al. demonstrated that there is a link between chronic caffeine intake to impaired cognitive recovery post-TBI injury [66]. There is a crucial need for further research to find an efficacious optimal dosage. Various caffeine-associated preclinical and clinical findings for this section are shown in Table 2 of this paper.

### 3.3. Effect of Caffeine on the Immune System in TBI

TBI has a high incidence of mortality and morbidity [37]. Caffeine, a widely consumed stimulant, has been found to possess neuroprotective benefits in degenerative neurological disorders [44,72]. Additionally, caffeine impacts various immune cells, such as lymphocytes [72], natural killer (NK) cells [73], mesenchymal stem cells, and neutrophils [74]. In this review paper, we will explore the effect of caffeine on the pathophysiology of an injured brain, its influence on immune cells, and its potential effects on dendritic changes in TBI.

Caffeine exerts neuroprotective effects and helps reduce neuroinflammation and oxidative stress: critical factors in the progression of neurodegenerative diseases [74]. A cohort study analyzed serum caffeine concentrations in TBI patients who presented to the emergency department (ED). Serum caffeine levels within 4 h were assessed and classified into three groups: low (0.01–0.58 μg/mL), intermediate (0.59–1.66 μg/mL), and high (1.67–10.00 μg/mL). The study found that individuals in the low- and intermediate-caffeine groups had a higher likelihood of functional recovery at 6 months compared to those in the no-caffeine group [44].

Caffeine’s antioxidant properties are key to protecting neuronal cells from damage induced by reactive oxygen species (ROS). Additionally, caffeine modulates inflammatory pathways, reducing the production of pro-inflammatory cytokines, which further contribute to its neuroprotective effects [64]. Caffeine also enhances synaptic plasticity and promotes neurogenesis, thereby aiding in cognitive recovery following TBI [66,74].

A study on adult female albino mice investigated caffeine’s impact on immune cells and tumor growth. Caffeine was shown to reduce the expression of PD-1 on cytotoxic T lymphocytes, which enhances their ability to target tumor cells. Mice treated with caffeine showed a significant decrease in tumor size, suggesting that caffeine could enhance anti-tumor immunity [72].

Caffeine’s effects on natural killer (NK) cells were also examined in a study involving 14 male cyclists. The cyclist ingested either 0, 2, or 6 mg/kg of caffeine before engaging in prolonged cycling. Blood samples were collected to assess the activation of NK cells in response to antigen stimulation post-exercise. Results revealed that both low (2 mg/kg) and high (6 mg/kg) doses of caffeine significantly enhanced NK cell activation compared to the placebo. Interestingly, no significant differences were observed between the two caffeine doses, indicating that even a low dose is effective in boosting immune function during prolonged physical activity [73].

In addition, a study analyzed the effects of caffeine on rats during development, focusing on how caffeine exposure impacts dendritic spine density in the prefrontal cortex and hippocampus, as well as behavioral and cognitive outcomes post-TBI. Rats exposed to caffeine during development exhibited altered spine density in both the prefrontal cortex and hippocampus. These structural changes were correlated with impaired memory and cognitive function, suggesting that caffeine consumption during development can significantly affect brain structure and function. Furthermore, caffeine-exposed rats displayed altered dendritic spine density and impaired cognitive function, as well as different recovery patterns from TBI, compared to controls, indicating that developmental caffeine exposure may have lasting effects on brain development and TBI recovery [48].

In conclusion, caffeine demonstrates significant neuroprotective and immune-modulating effects, which can aid recovery in TBI and offer potential benefits for neurodegenerative conditions. Both human and animal studies highlight caffeine’s positive impact on immune cells, cognitive function, and recovery post-TBI. However, further research is needed to fully understand the long-term implications and optimal dosing for therapeutic use. A summary of caffeine’s neuroprotective and immune-modulating properties is presented in Table 3 of this paper.

### 3.4. Effect of Caffeine on Various Physiological Proteins and Elements in TBI

Caffeine influences essential electrolytes, trace elements (iron), and specific amino acids, as well as proteins such as podoplanin and morphogens, particularly in the context of TBI. Understanding these interactions is crucial for managing TBI patients, where metabolic and neurochemical balance is critical for recovery. Caffeine can both affect and impair TBI recovery [75]. One pre-clinical study examined how acute versus chronic caffeine treatment affects brain injury in a mouse model of TBI [65]. The study showed that chronic caffeine treatment decreased glutamate release and inflammatory cytokine production, therefore helping to mitigate brain injury through the A1 receptor-mediated mechanism [65]. Another pre-clinical study investigated the short-term effects of caffeine on L-arginine metabolism in rat brains. It demonstrated that caffeine modulates L-arginine metabolism by decreasing arginase activity and increasing NO production without affecting TNF-α levels [76].

Oxidative stress, a significant factor in neurotoxicity in TBI and neurodegenerative diseases, arises from an imbalance between the production of free radicals, such as ROS, and the body’s antioxidant defenses. The association between aluminum (Al) exposure and oxidative stress is crucial for cognitive well-being, as Al exhibits strong prooxidant characteristics. A study designed an experimental model to investigate the effects of caffeinated coffee and caffeine on the survival of PC12 cells and the generation of ROS when exposed to neurotoxic agents such as aluminum maltolate (Almal) and/or hydrogen peroxide. It concluded that caffeinated coffee could prevent AML-induced neurotoxicity in PC12 cells, and caffeine resulted in a higher than fivefold increase in the survival rate of PC12 cells [77].

Caffeine has a diuretic effect, which can increase urine output [78]. This can lead to higher excretion of both sodium and potassium. A study found that high doses of caffeine significantly increased the urinary excretion of these electrolytes over 24 h [79]. The balance of sodium and potassium in the body is critical for nerve function and muscle contractions [78]. The Northern University, a tertiary care hospital in Thailand, found that, in TBI, post-operative death increased with high levels of blood glucose, hypernatremia, and acidosis [80], and hypokalemia was found to be the most common electrolyte imbalance [77]. A fundamental mechanism contributing to neuronal injury and death following TBI is the disruption of cellular calcium homeostasis, particularly involving intracellular calcium stores in the endoplasmic reticulum [81]. Using an in vitro model of stretch-induced traumatic injury and fura-2 digital calcium imaging, this study investigated changes in calcium-induced calcium release (CICR) and inositol (1,4,5)-trisphosphate (IP3)-linked signaling in cultured rat cortical neurons [77]. Caffeine, which stimulates CICR, caused a rapid increase in intracellular free calcium in 70% of uninjured neurons [76]. However, this response decreased to 30% fifteen minutes after injury. These alterations could affect normal neurotransmission in the brain and may contribute to some of the pathology of TBI [79].

### 3.5. How Does Caffeine Interfere with Concussion and TBI Recovery?

According to the National Institute of Neurological Disorders and Stroke (NINDS), TBI is defined as a form of acquired brain injury that occurs when a sudden trauma causes damage to the brain and can result when the head suddenly and violently hits an object or when an object pierces the skull and enters brain tissue [82]. The association between caffeine and its role in concussion and TBI recovery is contradictory. Pre-clinical studies have shown that chronic caffeine treatment can alleviate blast-induced TBI in mice [40] and that chronic caffeine exposure can reduce the effects of moderate blast wave-induced memory deficits in mice [39]. Another pre-clinical study on the effects of caffeine has also demonstrated that the inflammatory cell infiltration at a 24-h time point post-TBI was significantly reduced in mice pretreated chronically with 0.25 g/L caffeine in drinking water, suggesting a neuroprotective role of caffeine in TBI and, thus, concussion recovery [65]. Furthermore, serum caffeine level has been identified as a biomarker to determine the likelihood of a positive TBI outcome in some clinical studies [44].

The chronic effects of caffeine, investigated in a clinical study for post-TBI injury recovery, indicated that it might provide beneficial effects to neurological recovery but may also worsen outcomes [83]. Caffeine produces metabolites (theobromine, paraxanthine, and theophylline) that can be measured and are useful indicators to determine how it contributes to the pathophysiology and clinical outcomes of patients with TBI [35]. The timing of caffeine administration before or after TBI can affect the outcome. Chronic caffeine treatment after TBI can impair the recovery of motor function, further exacerbating the clinical outcomes of patients [38]. One of the mechanisms of how caffeine affects TBI recovery might be its effects on the brain’s microstructure in its gray and white matter regions [61], but further study is needed to understand how caffeine causes the dynamic change among brain structures and function. In conclusion, additional research is needed to recommend the amount of caffeine consumption that can be prescribed to patients with TBI. Various pre-clinical and clinical studies are summarized in Table 4 of this paper.

### 3.6. Effect of Preinjury and Post-Injury Exposure to Caffeine

The effects of caffeine in rats with TBI have been studied extensively, focusing on motor function, cognitive function, and mortality. In the 2012 article “Survival and Injury Outcome After TBI: Influence of Pre and Post-Exposure to Caffeine”, Sun et al. [84] demonstrated that pre-exposure to caffeine provided the most significant protective effect against mortality and injury severity following TBI. The study consisted of several phases assessing chronic pre-injury caffeine use, the impact of pre- and post-injury caffeine exposure, and the dose-dependency and efficacy of caffeine as a rescue treatment. The first phase examined rats with chronic caffeine use before TBI and caffeine-naive rats. The second phase monitored the effects of pre-injury caffeine use for three weeks and post-injury caffeine use. The third part investigated whether the caffeine rescue dosage was dose-dependent, the bolus’s efficacy, and possible negative effects. The findings indicated that pre-exposure to caffeine was most beneficial, with post-injury and continuous exposure also offering benefits, though to a lesser extent [84].

Further research by Lusardi et al. [37] in the same year examined the effects of caffeine on mortality and morbidity following TBI. In the article “Caffeine prevents acute mortality after TBI in rats without increased morbidity”, caffeine was administered intraperitoneally at doses comparable to human consumption levels, both before and after injury. The study monitored the rats’ mortality rates and assessed various behavioral and histological outcomes to evaluate morbidity. The results showed a significant reduction in acute mortality following TBI in caffeine-treated rats compared to untreated controls, with no increase in morbidity. Together, these studies underscore the potential neuroprotective benefits of caffeine in managing TBI [37]. While these studies highlight the acute benefits of caffeine, it is important to consider the chronic effects of caffeine on cognitive and motor functions, particularly in the context of TBI.

In the 2017 study, “Behavioral and Pathophysiological Outcomes Associated with Caffeine Consumption in RmTBI in Adolescent Rats”, Yamakawa et al. [64] explored these chronic effects. Adolescent rats were given either caffeinated or non-caffeinated water and subjected to three mild brain injuries. Behavioral assessments included tests for balance and motor coordination, anxiety-like behaviors, short-term working memory, and depressive-like behaviors. The findings revealed that chronic caffeine consumption during adolescence significantly altered normal developmental trajectories and the recovery process from repeated mild TBI. Behavioral differences were observed in balance, motor coordination, anxiety, memory, and depressive behaviors, which were influenced by the timing of caffeine exposure and the occurrence of injury. The study emphasizes the importance of monitoring caffeine consumption in adolescents at high risk for repeated mild TBI due to its significant effects on both behavioral and pathophysiological outcomes [64].

Building on these findings, in 2019, Sanjakdar et al. [66] examined the impacts of caffeine on motor and cognitive outcomes in rats with penetrating ballistic-like brain injury (PBBI). The study evaluated the effects of moderate (7%-PBBI) and severe (10%-PBBI) injuries on cognitive and motor functions, with rats receiving a 25 mg/kg caffeine bolus one hour before injury or no caffeine. The results indicated that caffeine-pre-treated rats exhibited improved motor recovery compared to controls. However, cognitive outcomes were worsened by caffeine pre-treatment, suggesting that chronic caffeine consumption before PBBI could enhance motor recovery but impair cognitive functions [66].

In 2020, Lusardi et al. [38] expanded their research on the impacts of caffeine on motor and cognitive outcomes in rats with TBI. The study assessed both acute and chronic effects of caffeine on mortality and morbidity following TBI. Rats received either chronic caffeine pre-exposure or an acute dose of caffeine immediately following the injury. The findings indicated that a single acute dose of caffeine administered immediately after TBI prevented lethal apnea, a significant cause of acute mortality, without negatively impacting motor performance following sublethal injuries. However, chronic caffeine treatment post-injury impaired the recovery of motor function. The study also demonstrated that pre-exposure to caffeine did not significantly impact acute and delayed outcome parameters. Importantly, the study revealed that caffeine withdrawal did not have detrimental effects on motor recovery, contrasting with the negative impact observed with chronic caffeine treatment post-injury. Overall, the study highlighted that the timing and duration of caffeine exposure are critical factors in its effects on TBI outcomes [38].

Most recently, in 2023, Johnson et al. [85] conducted an observational cohort study on the effects of caffeine on adolescents with mild TBI (mTBI). The study included 80 adolescent caffeine consumers (CAF+) and 40 non-caffeine consumers (CAF-), with a mean age of 15 years. Behavioral outcomes were assessed using the Rivermead Post-Concussion Symptoms Questionnaire (RPQ), the Depression subscale of the Beck Youth Inventories—Second Edition (BYI-D), and the Behavior Rating Inventory of Executive Function (BRIEF), along with heart rate variability (HRV) measures. Results showed that caffeine consumers had significantly higher RPQ scores for emotional health and sleep, greater somatic discomfort, and higher depressive symptoms compared to non-consumers. No significant differences were observed in executive function or HRV metrics between the groups. The study suggests that adolescent caffeine consumers with mTBI may experience poorer emotional health, sleep quality, and greater somatic discomfort, highlighting the need for careful management of caffeine consumption in this population [85].

Together, these studies provide a comprehensive understanding of the complex role caffeine plays in both acute and chronic settings following TBI. While acute caffeine administration can offer neuroprotective benefits and improve motor recovery, chronic consumption poses risks, particularly in developmental contexts such as adolescence. The findings underscore the importance of individualized caffeine consumption guidelines for individuals at risk of or recovering from TBI.

The effects of caffeine on the neuroimmune axis have also been studied. One 2023 study by Lin et al. [86] looked at ten healthy young adults who were not regular caffeine drinkers and used water-extraction-with-phase-contrast-arterial-spin-tagging (WEPCAST) MRI to assess the time dependence of BBB permeability to water before and following the ingestion of 200 mg caffeine. They also examined cerebral blood flow, venous oxygenation, and cerebral metabolic rate of oxygen. The researchers found that while caffeine ingestion led to a significant decrease in cerebral blood flow and venous oxygenation, it did not alter the permeability-surface-area product of the BBB to water, indicating that caffeine does not significantly change BBB permeability despite its vasoconstrictive effects [86]. More recently, Mahroo et al. (2025) [87] explored BBB permeability in 10 healthy regular caffeine drinkers. Using several measurements as a proxy for BBB permeability measurement, they found that there was a potential increase in permeability, supposedly in response to caffeine-induced decrease in cerebral blood flow [87]. Both studies contained small sample sizes and would require further research. However, their mixed results may again suggest the important variable of caffeine consumption chronicity.

## 4. Conclusions and Future Perspectives

TBI has a high incidence of mortality and morbidity [37]. Caffeine has been shown to play a role in TBI and works as an antagonist on the A2A receptor, potentially aiding recovery [72]. Caffeine has been found to possess neuroprotective benefits in degenerative neurological disorders [44]. Caffeine is a widely consumed stimulant [88]. It affects immune cells such as lymphocytes [72], natural killer cells [73], mesenchymal stem cells, and neutrophils [74]. In this review, we discuss the current knowledge on both the positive and negative effects of caffeine in TBI.

Caffeine exerts neuroprotective effects primarily through reducing neuroinflammation and oxidative stress, both of which are critical factors in neurodegenerative diseases. By inhibiting inflammatory pathways, caffeine reduces the production of pro-inflammatory cytokines, including TNF-α and IL-1β [64]. It promotes synaptic plasticity and neurogenesis, both essential for cognitive recovery, by enhancing brain-derived neurotrophic factor (BDNF) signaling, which has been shown to improve learning and memory [66,72].

Caffeine also affects natural killer (NK) cells and can boost immune function during physical stress, which could be beneficial for recovery following physical trauma such as TBI. Caffeine consumption can alter spine density, impair cognitive functions, cause different recovery patterns from TBI, and impact brain structure and function by modifying synaptic plasticity [48]. By combining evidence from human and animal studies, we were able to identify critical gaps in understanding the optimal dosage, timing, and long-term effects of caffeine in TBI recovery. These findings could inform clinical practices, such as dietary recommendations or adjunct treatments for patients recovering from TBI. Understanding the individual variability in caffeine metabolism and its influence on recovery is essential for personalized treatment approaches.

There are, however, a number of limitations to consider in the use of caffeine in TBI patients. However, acute caffeine administration immediately before or after TBI can exacerbate injury outcomes, including increased mortality, edema, disruption in the blood–brain barrier [40,47], and better long-term functional outcomes [44]. Several preclinical studies have demonstrated that caffeine can impair cognitive and motor recovery, particularly in models of repetitive mild TBI [48,63]. The effects of caffeine may also be influenced by sleep deprivation and the use of sedatives [61,89].

Furthermore, nano-caffeine represents a promising frontier in the advancement of therapeutic strategies for traumatic brain injury (TBI), offering enhanced drug delivery, targeted precision, and reduced systemic toxicity. By incorporating caffeine into nano-formulations such as liposomes, polymeric nanoparticles, dendrimers, and micelles. Researchers aim to improve its bioavailability, facilitate efficient penetration across the blood–brain barrier, and enable controlled release at sites of neural injury. These delivery platforms may potentiate caffeine’s neuroprotective properties, including the attenuation of oxidative stress, neuroinflammation, and excitotoxic damage, while mitigating the adverse effects associated with higher systemic doses. Furthermore, the co-delivery of nano-caffeine with anti-inflammatory agents or neurotropic factors may yield synergistic effects in promoting neural repair and regeneration. Notably, preclinical studies utilizing nano-coffee particles have demonstrated improvements in neuronal health, behavioral outcomes, and dendritic architecture in murine models of TBI, supporting the translational potential of this novel therapeutic approach [45]. Further investigations into targeted delivery, dosing kinetics, and long-term safety of nano-caffeine in both animal models and clinical trials are essential to validate its translational potential in acute and chronic TBI management.

This review emphasizes the critical need for continued research and innovation to establish standardized protocols for caffeine use in the therapeutic management of traumatic brain injury (TBI). As our understanding of caffeine’s neuroprotective effects evolves, it becomes increasingly important to explore its potential benefits and risks in diverse clinical contexts. The establishment of evidence-based guidelines will ensure that caffeine is used safely and effectively, maximizing its therapeutic potential while minimizing potential adverse effects. Such protocols should consider factors such as optimal dosing, timing, and patient demographics to tailor interventions to individual needs, paving the way for personalized medicine in TBI care.

Furthermore, a clearer understanding of caffeine’s role in TBI recovery will have broader implications for trauma care at both regional and national levels. By integrating the latest research findings into clinical practice, healthcare providers can improve the quality of trauma care, ensuring that treatment strategies are based on the best available evidence. This could lead to improved recovery rates, reduced complications, and more efficient management of TBI cases, ultimately influencing policy decisions, resource allocation, and the development of trauma care protocols. As a result, ongoing research into caffeine’s therapeutic effects in TBI not only holds promise for better patient outcomes but also represents a crucial step in advancing the field of trauma medicine and personalized healthcare.

## Figures and Tables

**Table 2:** This table summarizes various caffeine-associated preclinical and clinical findings in Traumatic Brain Injury (TBI).

Reference Number	Study Model	Findings
[35]	N/A	Caffeine acts as an adenosine antagonist, specifically at the A_1_ and A_2A_ receptors.
[67]	N/A	Adenosine receptors are of interest in TBI because they are involved in various brain injury pathways.
[36]	Rodent	Adenosine may have a protective role in the recovery of TBI
[22]	Humans	Caffeine may have a neuroprotective effect on Parkinson’s disease pathways.
[31]	Rodent	Caffeine may have a neuroprotective effect on neurodegenerative diseases
[36]	Rodent	Chronic caffeine treatment, rather than acute caffeine treatment, showed better recovery from TBI in mouse models.
[68]	Mixed	Review article finding combined evidence of potential benefit of treating Parkinson’s Disease and TBI through targeting the adenosine_2A_ receptor.
[65]	Rodent	Pretreatment of rats with caffeine before TBI showed an increase in mortality
[39]	Rodent	Chronic caffeine use may have some protective benefits in blast-induced TBI; however, chronic and acute caffeine use both increase mortalities.
[37]	Rodent	Caffeine injection given immediately after TBI reduced TBI-induced mortality in rat models.
[38]	Rodent	An acute dose of caffeine given 10 s after TBI decreased mortality and morbidity in mouse models.
[35]	Humans	An increase in CSF concentration of caffeine in patients with TBI was associated with more favorable outcomes.
[44]	Human	Patients with TBI who had a serum caffeine concentration between 0.01 to 1.66 μg/mL had better recovery after 6 months of injury compared to the no caffeine group.
[69]	Cats	One of the earliest studies that examined the pathophysiology of oxygen radicals in TBI
[70]	Humans	Found potential benefits of adenosine on cerebral blood flow and oxidative metabolism in patients with severe head injury
[48]	Rodent	Caffeine in female rats has been shown to decrease oxidative stress and specifically decrease lipid peroxidation in the hippocampus.
[71]	Rabbits	Caffeine was seen to protect against oxidative stress and Alzheimer’s dementia-like pathology in rabbit models.
[45]	Rodent	Positive neuronal changes were seen when treating mice with nano coffee injections after a TBI.

N/A—not available.

**Table 3:** This table summarizes the effects of caffeine on TBI, including its neuroprotective and immune-modulating properties. Further research is necessary to fully understand the long-term implications and optimal dosing for therapeutic use.

Study/Experiment	Details
TBI Mortality and Morbidity	High incidence of mortality and morbidity in TBI patients [37]
Caffeine Neuroprotective Effects	Caffeine has neuroprotective benefits in degenerative neurological disorders, antagonizes A2A receptors [33]
Serum Caffeine in TBI Patients	Caffeine levels were analyzed within 4 h of injury and categorized into low, intermediate, and high levels; a higher likelihood of 6-month recovery in low- and intermediate-caffeine groups [44]
Caffeine Antioxidative Properties	Caffeine protects neuronal cells from ROS damage, modulates inflammatory responses, and lowers pro-inflammatory cytokine production [64,72]
Impact on Cytotoxic T Lymphocytes	Caffeine reduced PD1 expression on cytotoxic T lymphocytes, enhanced tumor targeting, and decreased tumor size [72]
Effect on Natural Killer Cells	Caffeine enhanced NK cell activation post-exercise in cyclists, effective at low and high doses [73]
Developmental Exposure in Rats	Developmental caffeine exposure in rats altered spine density, impaired memory and cognitive function, and different TBI recovery patterns [48]

**Table 4:** This table summarizes various pre-clinical and clinical studies of how caffeine interferes with concussion and TBI recovery.

Type of Study	Caffeine Treatment	Conclusion
Pre-Clinical Study	0.25 g/L	Chronic caffeine treatment alleviated cerebral injury at 24 h post-severe blast-induced TBI (bTBI) [39]
Pre-Clinical Study	5 mg/kg, 15 mg/kg and 50 mg/kg	Chronic caffeine treatment represses the release of glutamate and inhibits cytokine expression after TBI [65]
Clinical Study	0.01–10.00 μg/mL	A statistically significant association was found between a serum caffeine concentration of from 0.01 to 1.66 μg/mL and good functional recovery at 6 months after injury, compared with the no-caffeine group of patients with TBI with intracranial injury [44]
Pre-Clinical Study	Oral bolus dose of 25 mg/kg	Regular caffeine consumption before a penetrating brain injury may moderately improve motor recovery but worsen the neurocognitive sequelae associated with a penetrating brain injury [66]
Clinical Study	≥1 μmol/L (194 ng/mL)	Caffeine may be neuroprotective by long-term upregulation of adenosine A1 receptors or acute inhibition of A2a receptors [35]
Pre-Clinical Study	20 mg/kg	Intracranial pressure decreased by 11% from the baseline value, which can improve clinical outcomes post-TBI [83]
Pre-Clinical Study	25 mg/kg	Chronic treatment initiated after TBI suggested improved motor function with a nonspecific adenosine receptor agonist, but a slight decrease in motor function after an A1 receptor antagonist [47]
Pre-Clinical Study	36 mg/kg	The interventions of caffeine, sleep deprivation, sleep aids, and sedation during the acute post-mTBI period each changed the subclinical characteristics of the brain after mTBI and altered the return toward normal function [61].

## Data Availability

Information for this review was collected from various sources, including PubMed, Web pages, Google Scholar, Scopus, Web of Science, and ClinicalTrials.gov.
